# Implication of ARID1A Undercurrents and PDL1, TP53 Overexpression in Advanced Gastric Cancer

**DOI:** 10.3389/pore.2021.1609826

**Published:** 2021-12-03

**Authors:** Jasiya Qadir, Sabhiya Majid, Mosin Saleem Khan, Fouzia Rashid, Mumtaz Din Wani, Showkat Ahmad Bhat

**Affiliations:** ^1^ Department of Biochemistry, Government Medical College Srinagar and Associated Hospitals, Srinagar, India; ^2^ Department of Clinical Biochemistry, University of Kashmir, Srinagar, India; ^3^ Department of Surgery, Government Medical College Srinagar and Associated Hospitals, Srinagar, India; ^4^ Department of Biochemistry, Government Medical College, Doda, India

**Keywords:** gastric cancer, *ARID1A*, mutation, mRNA expression 3, mRNA expression, qRT-PCR, *TP53*, *PDL1*

## Abstract

AT-rich interactive domain-containing protein 1A (ARID1A), TP53 and programmed cell death-ligand 1 (PDL1) are involved in several protein interactions that regulate the expression of various cancer-related genes involved in the progression of the cell cycle, cell proliferation, DNA repair, and apoptosis. In addition, gene expression analysis identified some common downstream targets of ARID1A and TP53. It has been established that tumors formed by *ARID1A*-deficient cancer cells exhibited elevated *PDL1* expression. However, the aberrations in these molecules have not been studied in this population especially in Gastric Cancer (GC). In this backdrop we aimed to investigate the role of the *ARID1A* mutation and expression of *ARID1A*, *TP53* and *PDL1* genes in the etiopathogenesis of Gastric Cancer (GC) in the ethnic Kashmiri population (North India). The study included 103 histologically confirmed GC cases. The mutations, if any, in exon-9 of *ARID1A* gene was analysed by Polymerase Chain Reaction (PCR) followed by Sanger sequencing. The mRNA expression of the *ARID1A*, *TP53* and *PDL1* genes was analysed by Quantitative real time-PCR (qRT-PCR). We identified a nonsense mutation (c.3219; C > T) in exon-9 among two GC patients (∼2.0%), which introduces a premature stop codon at protein position 1073. The mRNA expression of the *ARID1A, TP53* and *PDL1* gene was significantly reduced in 25.3% and elevated in 47.6 and 39.8% of GC cases respectively with a mean fold change of 0.63, 2.93 and 2.43. The data revealed that reduced mRNA expression of *ARID1A* and elevated mRNA expression of *TP53* and *PDL1* was significantly associated with the high-grade and advanced stage of cancer. Our study proposes that *ARAD1A* under-expression and overexpression of *TP53* and *PDL1* might be crucial for tumor progression with *TP53* and *PDL1* acting synergistically.

## Introduction

Gastric cancer (GC) is prevalent and account for a large number of cancer deaths globally. Although there are considerable advances in cancer diagnosis and therapy, GC remains an important cancer worldwide and is responsible for over one million new cases in 2020 and an estimated 769,000 deaths, ranking fifth for incidence and fourth for mortality globally [1]. In Kashmir valley (North India), GC has been reported as the most frequently diagnosed cancer with an occurrence of around 18.8% among all cancer cases [2].

Multiple genetic and epigenetic alterations in oncogenes and tumor suppressor genes are involved in the process of gastric carcinogenesis [3]. The *ARID1A* gene encodes a component of the switch/sucrose non-fermentable (SWI-SNF) chromatin remodelling complex [4]. ARID1A is a helix-turn-helix, nucleocytoplasmic protein of approximately 250 kDa, whose stability varies according to its subcellular location [5]. *ARID1A* gene expression is down-regulated in S and G_2_/M phases and is up-regulated in the G_0_/G_1_ phase, which supports the role of *ARID1A* at the G_1_ checkpoint for the proper arrest of cell cycle progression [6]. ARID1A is involved in the modulation of various cellular processes that are vital in preventing tumor initiation and progression *via* regulating the downstream transcriptional activity of several proto-oncogenes and tumor suppressor genes (TSGs) [7]. *ARID1A* gene possesses a high frequency of somatic mutation in several types of malignancies leading to reduced or loss of expression, which in turn exhibits a positive correlation with tumorigenicity [6, 8]. Limited studies have evaluated the possible role of *ARID1A* so far. Loss of *ARID1A* expression was reported in 11–51.3% of GCs and related to poor clinical parameters and shorter survival of GC patients [9-10].

*TP53* is a well-studied tumor suppressor gene that plays a key role in regulating the cell cycle. It is a principal mediator of growth arrest, senescence and apoptosis in response to a broad array of cellular damage [11]. Interestingly, The SWI/SNF complex interacts directly or indirectly with TP53 and regulates the transcription of target genes downstream of *TP53*, thereby suggesting that *ARID1A* plays important roles in tumor suppression [4, 12]. Some studies have reported that mutation and aberrant expression of *TP53* are negatively associated with *ARID1A* loss [13].

PDL1, a molecule able to down-regulate immune response, is thought to play an important role in the persistence of chronic infections and evasion of immune destruction by tumor cells [14]. PD1 acts as a T-cells inhibitor mainly by limiting T-cells activity within neoplastic tissues and its ligand, PDL1, is often overexpressed on tumor cells [15]. As per previous studies, *PDL1* was overexpressed in various cancers including GC [16-18]. However, the prognostic relevance of PDL1 protein expression in GC remains controversial, and prior studies have shown that PDL1 plays a promotive or suppressive role in GC [19]. It was also reported that ARID1A expression is related to PDL1 levels in various cancers [16-18].

At present, the outcome for patients with advanced GC is still bleak [20]. Efforts have been directed toward identifying novel biomarkers for aggressive disease and new molecular targets for therapeutic intervention. Therefore, in this study, we investigated the mutation spectrum on exon-9 and expression of *ARID1A* along with expression of TP53 and PDL1 genes in GC. Furthermore, we also correlate the expression with various clinicopathological parameters to have an idea about the role of *ARID1A* in the genesis of GC.

## Materials and Methods

### Study Design

This was a cross-sectional study conducted by the Department of Biochemistry and General Surgery, Government Medical College Srinagar and Associated Shri Maharaja Hari Singh (SMHS) and Superspeciality Hospital, Srinagar, Kashmir, J&K, India.

### Study Subjects and Sample Collection

The study included histopathologically confirmed 103 Gastric tumor tissue samples along with their adjacent normal tissue collected from the Department of Surgery, Shri Maharaja Hari Singh (SMHS) Hospital, Srinagar from March 2017 to March 2020. Adjacent normal tissue contained normal gastric mucosa present near the margins of resection, away from the tumor. The clinicopathological information of the patients was obtained from the Medical Records Department of hospital. All the GC cases were newly diagnosed intestinal type adenocarcinomas and did not receive any chemo or radiotherapy. The included GC cases were not having any other type of tumour and were free from any genetic disorder. One aliquot of tissue sample was snap-frozen immediately and stored at −80°C till further processing for DNA analysis. Another aliquot of tissue sample was immediately stored in *RNA-later* (Sigma-Aldrich, United States) at 4°C overnight, to allow the solution to thoroughly penetrate the tissue before stored at −80°C to prevent any degradation until RNA isolation.

### DNA Isolation

DNA was extracted from tissue samples using *QIAamp DNA Mini kit* (Qiagen, Germany) according to the given protocol. The quality of the DNA was verified using 1% agarose gel electrophoresis. The concentration and purity of DNA was measured using *NanoDrop 2000c Spectrophotometer* (ThermoFisher Scientific, United States). The DNA samples of high molecular weight, without any fragmentation/shearing, with OD (260/280) ratio between 1.8 and 1.9 were processed for further molecular analysis.

### Polymerase Chain Reaction Followed by DNA Sequencing

Exon-9 of *ARID1A* gene was amplified using primers: forward: 5′CAC​AGC​ACT​ATT​TGG​CTC​CAG-3′; reverse: 5′-ATC​ATC​TCT​GGG​CTG​GCT​G-3′ (Eurofins Genomics, Germany). The PCR amplification was carried out in a 50 µl volume containing 12.5 µl of 2X PCR master mix (3B BlackBio, Biotech, India), 0.2 µM of each forward and reverse primers, 50–150 ng of genomic DNA. After initial denaturation at 94°C for 7 min, 35 cycles of denaturation at 94°C for 20 s, annealing at 58°C for 30 s, and extension at 72°C for 30 s were performed. The final extension was given at 72°C for 7 min. The 343 bp amplified product was verified on 2.5% agarose gel and visualized on *Omega Lum G* Gel Documentation centre (Aplegen, United States).

### DNA Sequencing

The amplified samples were sequenced, using *ABI prism 310* automated DNA sequencer (ThermoFisher Scientific, United States) *via* Sanger dideoxy method.

### RNA Isolation Followed by cDNA Synthesis

Total RNA was extracted from tissue samples using TRIzol reagent (ThermoFisher Scientific, United States). The concentration and purity of RNA was measured using *NanoDrop 2000c Spectrophotometer* (ThermoScientific, United States). RNA having A260/A280 between 1.8 and 2.0 was considered as “Uncontaminated.” Furthermore, RNA integrity was also verified by the presence of 28S, 18S and 5S rRNA bands on 1.5% agarose gel. The first strand cDNA was synthesized with DNase-treated RNA; 1–2 µg RNA was reverse transcribed into cDNA using *RevertAid First Strand cDNA Synthesis Kit* (ThermoFisher Scientific, United States), according to the manufacturer’s instructions. The reactions were incubated for 60 min at 37°C followed by 95°C for 10 min.

### Quantitative Real Time-PCR for Relative mRNA Expression of ARID1A, TP53 and PDL1

qRT-PCR was performed using *7500 Real-Time PCR system* (ThermoFisher Scientific, United States). The primers sequences used were; *ARID1A forward 5*′*-CTT​CAA​CCT​CAG​TCA​GCT​CCC​A-3′, ARID1A reverse 5′-GGT​CAC​CCA​CCT​CAT​ACT​CCT​TT-3*′*; TP53 forward 5′-TGC​GTG​TGG​AGT​ATT​TGG​ATG-3′, TP53 reverse 5′-TGG​TAC​AGT​CAG​AGC​CAA​CCT​C-3′; PDL1 forward 5′-ACT​GGC​ATT​TGC​TGA​ACG​CA-3′, PDL1 reverse 5′- AGA​CAA​TTA​GTG​CAG​CCA​GGT​CT-3′; GAPDH forward 5′- CTC​CTC​CTG​TTC​GAC​AGT​CAG​C-3; GAPDH reverse, 5′- CCC​AAT​ACG​ACC​AAA​TCC​GTT-3*′ [10, 21–22]. The *GAPDH* (housekeeping gene) was used as an internal control. The PCR reaction mixture contained 10 µl of *KAPA SYBR® FAST master mix* (Sigma-Aldrich, United States), 0.5 µl of cDNA of each sample, 0.2 µM of each forward and reverse primers in a final volume of 20 µl adjusted with Milli-Q water. The reaction mix was preheated at 95°C for 10 min and then amplified with 40 cycles at 95°C for 30 s, X°C for 1 min (X = 60°C for ARID1A; 65°C for TP53, 56°C for PDL1) and 72°C for 35 s. Specificity of the PCR products was determined by Melting curve analysis. All the samples were run in triplicates. The mRNA expression of *ARID1A* was defined on the basis of C_t_ (cycle threshold) value of each sample. The relative expression was calculated by the 2^-∆∆Ct^ method [23].

### Statistical Analysis

Data analysis was performed using SPSS software V 23.0 (SPSS Inc., Chicago IL, United States). The association between *ARID1A, TP53, PDL1* alterations and various sociodemographic and clinicopathological characteristics was evaluated by Pearson’s χ^2^ test or Fisher’s exact test for discrete variables; paired t-test for continuous variables using multiple logistic regression analysis. The odds ratios (ORs) with 95% confidence intervals (CIs) was calculated. Two-sided *p* ≤ 0.05 was considered statistically significant.

## Results

### Patient Characteristics

Out of all GC patients taken for the study 59.3% (61 of 103) were males whereas 40.7% (42 of 103) were females. The mean age (in years) of GC cases was 56.6 ± 12.1. The mean BMI in kg/m^2^ of cases was 24.85 ± 4.45, while as the mean CEA levels of GC cases was 6.07 ± 2.04 ng/ml 57.3% of GC cases were non-smokers and 42.7% were smokers. *H. Pylori* status was positive in 36.9% (38 of 103) of GC patients. The detailed socio-demographic and clinicopathological parameters of GC patients are given in Table 1.

**TABLE 1:** Socio-demographic and clinicopathological variables of GC patients taken for the study.

Variables	GC cases (n = 103)	%
Gender		
Male	61	59.3
Female	42	40.7
Age group		
<50 years	35	34.0
≥50 years	68	66.0
Dwelling		
Rural	66	64.0
Urban	37	36.0
Smoking status		
Non-Smoker	59	57.3
Smoker	44	42.7
BMI (kg/m^2^)		
Normal	54	52.4
Underweight	10	9.7
Preobese	28	27.2
Obese Class I	09	8.7
Obese Class II	02	1.9
Family history		
No	86	83.5
Yes	17	16.5
Salt tea consumption		
<5 cups/day	29	28.2
≥5 Cups/day	74	71.8
CEA levels (ng/ml)		
Normal	34	33.0
Elevated	69	67.0
H. Pylori	65	63.1
Absent Present	38	36.9
Stage		
I and II	70	68.0
III and IV	33	32.0
Grade		
WD	66	64.0
PD	37	36.0

BMI, basal metabolic index (<18.5 = underweight, 18.5–24.99 = Normal, 25–29.99 = Preobese, 30–34.99 = Obese class I, 35–39.99 = Obese class II).

CEA, carcinoembryonic antigen; *H. Pylori*, *Helicobacter pylori*; WD, well differentiated; PD, poorly differentiated.

### Mutational Analysis of ARID1A Gene

**FIGURE 1:**
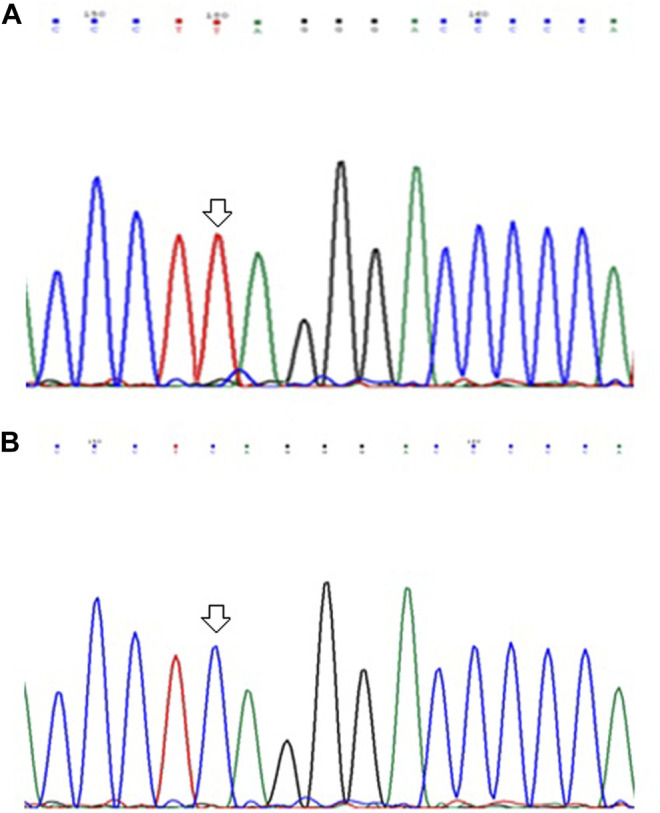
Partial electrophoretograms (forward) of DNA sequences in exon-9 of *ARID1A* gene in GC cases showing **(A)** C to T substitution at nucleotide position 3219 and **(B)** Sequence without any mutation.

In the present study, Gastric tumor tissue samples were screened along with their adjacent non-tumor tissue samples for the presence of mutations, if any. On DNA sequencing of amplified exon-9 of *ARID1A* gene, we detected a nonsense mutation (c. 3219 C > T) at amino acid position 1073, among 02 out of 103 (∼2.0%) GC tumor tissues samples leading to formation of stop codon (CAG to TAG). Mutation was not found in any of the adjacent normal tissue samples. Figure 1 shows the partial electrophoretograms depicting the c.3219; C > T mutation in exon-9 of *ARID1A* gene.

### Relative mRNA Expression of ARID1A, TP53, PDL1 in GC

**FIGURE 2:**
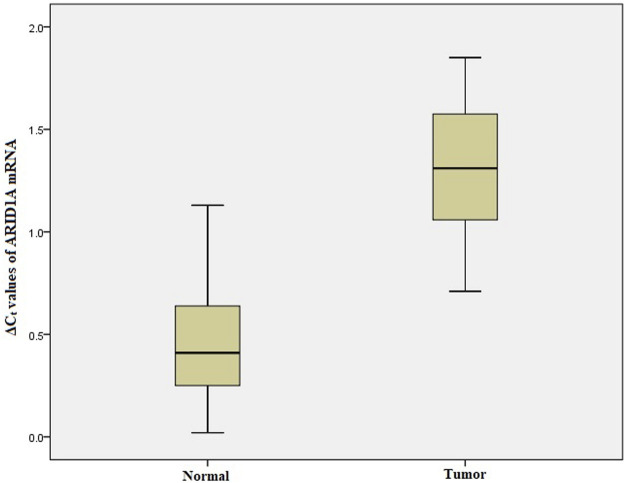
Box and whisker plot depicting the relative mRNA expression of *ARID1A* in terms of ∆Ct values of GC tumor tissues and adjacent normal tissues (controls). The experiment was performed in triplets. The relative mRNA expression of ARID1A was significantly lower in Gastric tumors tissue samples compared to adjacent non-tumorous tissues (*p* < 0.001). Data was represented as mean ± SD.

**FIGURE 3:**
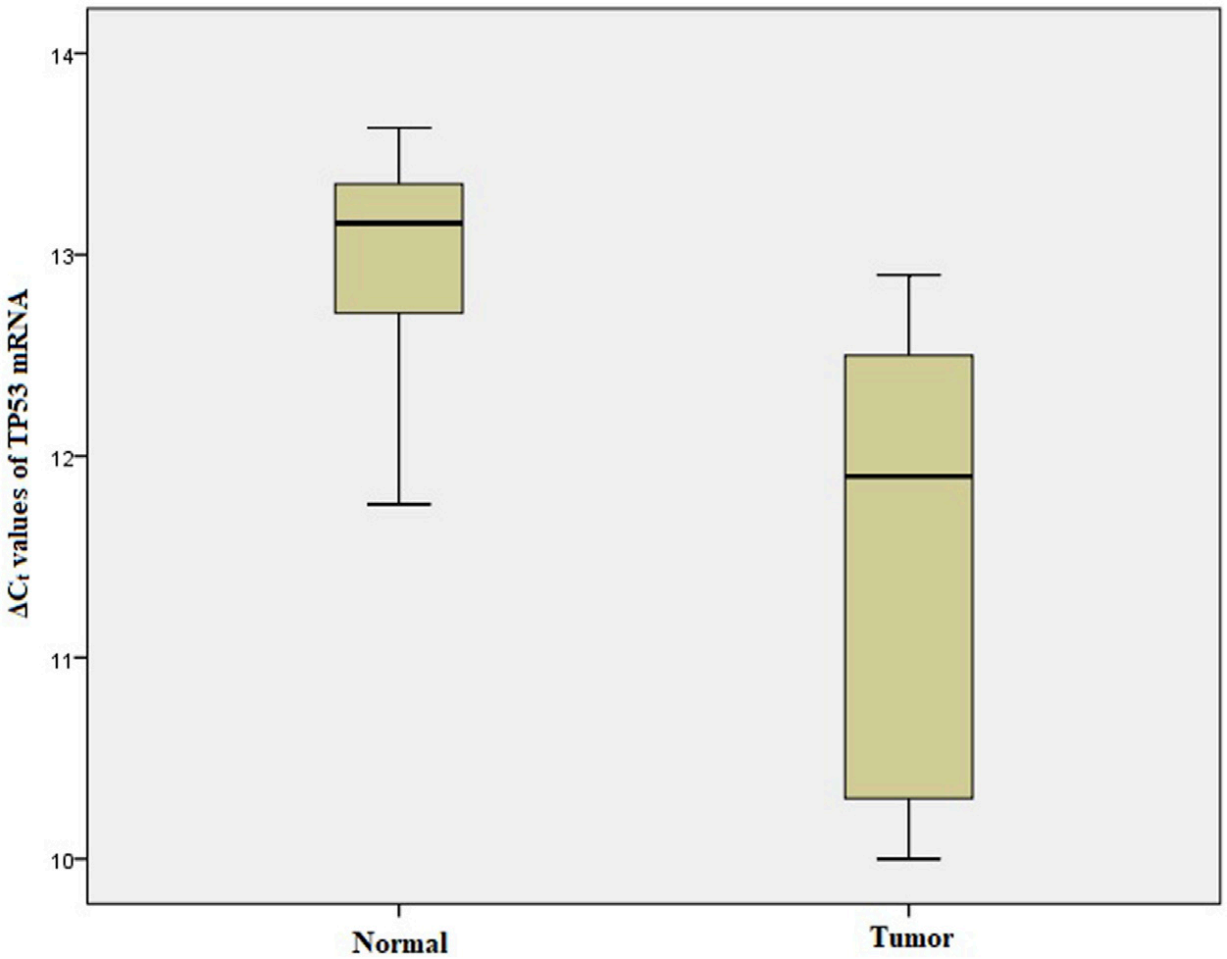
Box and whisker plot depicting the relative mRNA expression of *TP53* in terms of ∆Ct values of GC tumor tissues and adjacent normal tissues (controls). The experiment was performed in triplets. The relative mRNA expression of TP53 was significantly lower in Gastric tumors tissue samples compared to adjacent non-tumorous tissues (*p* < 0.001). Data was represented as mean ± SD.

**FIGURE 4:**
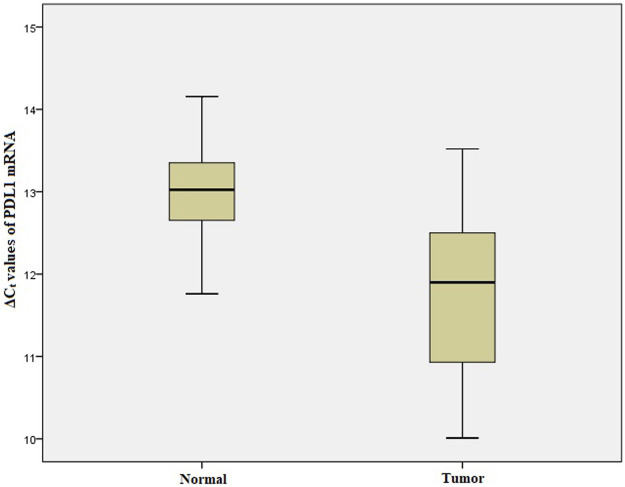
Box and whisker plot depicting the relative mRNA expression of *PDL1* in terms of ∆Ct values of GC tumor tissues and adjacent normal tissues (controls). The experiment was performed in triplets. The relative mRNA expression of PDL1 was significantly lower in Gastric tumors tissue samples compared to adjacent non-tumorous tissues (*p* < 0.001). Data was represented as mean ± SD.

We performed qRT-PCR to investigate *ARID1A, TP53* and *PDL1* mRNA expression in 103 Gastric tumor tissues and their adjacent normal tissue samples. The melt curve analysis showed zero formation of any non-specific products. Figures 2–4 contains box and whisker plots depicting the relative mRNA expression of *ARID1A, TP53 and PDL1* in GC cases in terms of their **Δ**C_t_ values. There was a significant decrease in the mRNA expression of *ARID1A* in Gastric tumor tissues compared to adjacent normal tissues (∆C_t_ tumor vs. ∆C_t_ adjacent normal; *p* < 0.0001; Table 2) with a mean fold change of 0.63. In addition, there was a significant increase in the mRNA expression of *TP53* and *PDL1* in Gastric tumor tissues compared to adjacent normal tissues (∆C_t_ tumor vs. ∆C_t_ adjacent normal; *p* < 0.0001; Table 2) with a mean fold change of 2.93 and 2.43 respectively.

**TABLE 2:** Comparison of mRNA expression of *ARID1A, TP53 and PDL1* gene between GC tumor tissues and adjacent normal tissues.

Gene	Gastric tumor tissues ΔC_t_ (mean ± SD)	Adjacent normal tissue ΔC_t_ (mean ± SD)	Mean fold change (2^−ΔΔCT^)	*p*-Value
*ARID1A*	1.3 ± 0.34	0.53 ± 0.38	0.63	<0.0001
*TP53*	11.45 ± 1.0	13.0 ± 0.42	3.93	<0.0001
*PDL1*	11.63 ± 0.97	12.91 ± 0.5	3.2	<0.0001

In the present study, “increased” mRNA expression was defined as N-fold ≥2.0, “normal” expression was an N-fold ranging from 0.5001 to 1.9999, and “decreased” mRNA expression was N-fold ≤0.5 [24]. Using this criteria, the relative mRNA expression of the *ARID1A* was decreased in 25.24% (26 of 103) of GC patients (Table 3). The relative mRNA expression of *TP53* and *PDL1* was increased in 47.6% (49 of 103) and 39.8% (41 of 103) of GC patients respectively (Tables 4, 5).

**TABLE 3:** Association of *ARID1A* mRNA expression with socio-demographic and clinicopathological variables of GC patients.

Variables	Cases N = 103 (%)	*ARID1A* mRNA expression		OR (95% CI)	*p*-Value
		Normal 77 (74.8%)	Reduced 26 (25.2%)		
Gender					
Male	61 (59.)	42 (68.8)	19 (31.2)	1.00	0.10
Female	42 (40.7)	35 (83.3)	07 (16.7)	0.4 (0.15–1.6)	
Age group					
<50 years	35 (34.0)	28 (80.0)	07 (20.0)	1.00	0.40
≥50 years	68 (66.0)	49 (72.0)	19 (28.0)	1.5 (0.6–4.4)	
Dwelling					
Rural	66 (64.0)	51 (77.2)	15 (22.8)	1.00	0.44
Urban	37 (36.0)	26 (70.2)	11 (29.8)	1.4 (0.56–3.6)	
Smoking status					
Non-Smoker	59 (57.3)	47 (80.0)	12 (20.0)	1.00	0.20
Smoker	44 (42.7)	30 (68.1)	14 (31.9)	1.8 (0.73–4.5)	
BMI (kg/m^2^)					
Normal	54 (52.4)	41 (76.0)	13 (24.0)	1.00	
Underweight	10 (9.7)	06 (60.0)	04 (40.0)	2.1 (0.45–8.8)	0.3
Preobese	28 (27.2)	22 (78.5)	06 (21.5)	0.9 (0.27–2.6)	0.8
Obese Class I	09 (8.7)	06 (66.7)	03 (33.3)	1.6 (0.3–7.2)	0.5
Obese Class II	02 (1.9)	02 (100.0)	00 (0.0)	1.0 (0.03–10.2)	0.9
Family history					
No	86 (83.5)	65 (75.5)	21 (24.5)	1.00	0.66
Yes	17 (16.5)	12 (70.5)	05 (29.5)	1.3 (0.37–4.0)	
Salt tea consumption					
<5 cups/day	29 (28.2)	19 (65.5)	10 (34.5)	1.00	0.19
≥5 Cups/day	74 (71.8)	58 (78.3)	16 (21.7)	0.53 (0.2–1.4)	
CEA levels (ng/ml)					
Normal	34 (33.0)	23 (67.6)	11 (32.4)	1.00	0.25
Elevated	69 (67.0)	54 (78.2)	15 (21.8)	0.58 (0.2–1.4)	
*H. Pylori*					
Absent	65 (63.1)	51 (78.4)	14 (21.6)	1.00	0.27
Present	38 (36.9)	26 (68.4)	12 (31.6)	1.7 (0.66–4.2)	
Stage					
I and II	70 (68.0)	57 (81.4)	13 (18.6)	1.00	0.03
III and IV	33 (32.0)	20 (60.6)	13 (39.4)	2.8 (1.1–7.2)	
Grade					
WD	66 (64.0)	54 (81.8)	12 (18.2)	1.00	0.03
PD	37 (36.0)	23 (62.1)	14 (37.9)	2.7 (1.1–6.9)	
*TP53* mRNA expression					
Normal	54 (52.4)	39 (72.2)	15 (27.8)	1.00 (Ref.)	0.6
Reduced	49 (47.6)	38 (77.5)	11 (22.5)	0.7 (0.3–1.8)	
*PDL1* mRNA expression					
Normal	62 (60.2)	46 (74.1)	16 (25.8)	1.00 (Ref.)	0.9
Elevated	41 (39.8)	31 (75.6)	10 (24.4)	0.9 (0.3–2.3)	

BMI, basal metabolic index (<18.5 = underweight, 18.5–24.99 = Normal, 25–29.99 = Preobese, 30–34.99 = Obese class I, 35–39.99 = Obese class II).

CEA, carcinoembryonic antigen; *H. Pylori*, *Helicobacter pylori*; WD, well differentiated; PD, poorly differentiated.

**TABLE 4:** Association of *TP53* mRNA expression with socio-demographic and clinicopathological variables of GC patients.

Variables	Cases N = 103 (%)	*TP53* mRNA expression		OR (95% CI)	*p*-Value
		Normal 54 (52.4)	Elevated 49 (47.6)		
Gender					
Male	61 (59.3)	29 (47.5)	32 (52.5)	1.00 (Ref.)	0.3
Female	42 (40.7)	25 (59.5)	17 (40.5)	0.6 (0.3–1.4)	
Age group					
<50 years	35 (34.0)	21 (60.0)	14 (40.0)	1.00 (Ref.)	0.3
≥50 years	68 (66.0)	33 (48.5)	35 (51.5)	1.6 (0.7–3.6)	
Dwelling					
Rural	66 (64.0)	37 (56.1)	29 (43.9)	1.00 (Ref.)	0.4
Urban	37 (36.0)	17 (45.9)	20 (54.1)	1.5 (0.7–3.3)	
Smoking status					
Non-Smoker	59 (57.3)	36 (59.0)	25 (41.0)	1.00 (Ref.)	0.1
Smoker	44 (42.7)	18 (42.9)	24 (57.1)	1.9 (0.8–4.2)	
BMI (kg/m^2^)					
Normal	54 (52.4)	37 (68.5)	17 (31.5)	1.00 (Ref.)	
Underweight	10 (9.7)	05 (50.0)	05 (50.0)	2.1 (0.5–9.0)	0.2
Preobese	28 (27.2)	07 (32.1)	21 (67.9)	6.3 (2.3–18.9)	**0.0002**
Obese Class I	09 (8.7)	04 (66.7)	05 (33.3)	2.6 (0.6–12.5)	0.2
Obese Class II	02 (1.9)	01 (100.0)	01 (0.0)	2.1 (0.05–8.7)	0.6
Family history					
No	86 (83.5)	47 (53.4)	41 (46.6)	1.00 (Ref.)	0.7
Yes	17 (16.5)	07 (46.7)	08 (53.3)	1.3 (0.4–3.9)	
Salt tea consumption					
<5 cups/day	29 (28.2)	19 (65.5)	10 (34.5)	1.00 (Ref.)	0.1
≥5 Cups/day	74 (71.8)	35 (47.3)	39 (52.7)	2.1 (0.8–5.3)	
CEA levels (ng/ml)					
Normal	34 (33.0)	37 (53.6)	32 (46.4)	1.00 (Ref.)	0.8
Elevated	69 (67.0)	17 (50.0)	17 (50.0)	1.2 (0.5–2.6)	
*H. Pylori*					
Absent	65 (63.1)	42 (64.6)	23 (35.4)	1.00 (Ref.)	**0.002**
Present	38 (36.9)	12 (31.6)	26 (68.4)	4.0 (1.7–9.2)	
Stage					
I and II	70 (68.0)	44 (62.0)	27 (38.0)	1.00 (Ref.)	**0.005**
III and IV	33 (32.0)	10 (31.3)	22 (68.8)	3.6 (1.5–8.7)	
Grade					
WD	66 (64.0)	42 (65.6)	22 (34.4)	1.00 (Ref.)	**0.001**
PD	37 (36.0)	12 (30.8)	27 (69.2)	4.3 (1.8–10.0)	
*PDL1* mRNA expression					
Normal	62 (60.2)	44 (71.0)	18 (29.0)	1.00 (Ref.)	**0.00**
Elevated	41 (39.8)	10 (24.4)	31 (75.6)	7.6 (3.1–18.6)	

BMI, basal metabolic index (<18.5 = underweight, 18.5–24.99 = Normal, 25–29.99 = Preobese, 30–34.99 = Obese class I, 35–39.99 = Obese class II).

CEA, carcinoembryonic antigen; *H. Pylori*, *Helicobacter pylori*; WD, well differentiated; PD, poorly differentiated.

**TABLE 5:** Association of *PDL1* mRNA expression with socio-demographic and clinicopathological variables of GC patients.

Variables	Cases N = 103 (%)	*PDL1* mRNA expression		OR (95% CI)	*p*-Value
		Normal 62 (60.2)	Elevated 41 (39.8)		
Gender					
Male	61 (59.3)	36 (59.0)	25 (41.0)	1.00 (Ref.)	0.8
Female	42 (40.7)	26 (61.9)	16 (38.9)	0.8 (0.4–2.0)	
Age group					
<50 years	35 (34.0)	22 (62.9)	13 (37.1)	1.00 (Ref.)	0.8
≥50 years	68 (66.0)	40 (58.8)	28 (41.2)	1.2 (0.5–2.7)	
Dwelling					
Rural	66 (64.0)	42 (63.6)	24 (36.4)	1.00 (Ref.)	0.4
Urban	37 (36.0)	20 (54.1)	17 (45.9)	1.5 (0.6–3.4)	
Smoking status					
Non-Smoker	59 (57.3)	45 (73.8)	16 (26.2)	1.00 (Ref.)	0.001
Smoker	44 (42.7)	17 (40.5)	25 (59.5)	4.1 (1.8–9.6)	
BMI (kg/m^2^)					
Normal	54 (52.4)	36 (66.7)	18 (33.3)	1.00 (Ref.)	
Underweight	10 (9.7)	09 (90.0)	01 (10.0)	0.2 (0.009–1.5)	0.1
Preobese	28 (27.2)	13 (46.4)	15 (53.6)	2.2 (0.9–5.9)	0.08
Obese Class I	09 (8.7)	03 (33.3)	06 (66.7)	3.9 (0.8–21.1)	0.07
Obese Class II	02 (1.9)	01 (50.0)	01 (50.0)	1.9 (0.04–8.1)	0.6
Family history					
No	86 (83.5)	51 (58.0)	37 (42.0)	1.00 (Ref.)	0.4
Yes	17 (16.5)	11 (73.3)	04 (26.7)	0.5 (0.1–1.7)	
Salt tea consumption					
<5 cups/day	29 (28.2)	20 (70.0)	09 (30.0)	1.00 (Ref.)	0.2
≥5 Cups/day	74 (71.8)	42 (56.7)	32 (43.3)	1.68 (0.7–4.3)	
CEA levels (ng/ml)					
Normal	34 (33.0)	42 (60.9)	27 (39.1)	1.00 (Ref.)	0.9
Elevated	69 (67.0)	20 (58.8)	14 (41.2)	1.1 (0.5–2.5)	
*H. Pylori*					
Absent	65 (63.1)	44 (67.7)	21 (32.3)	1.00 (Ref.)	0.06
Present	38 (36.9)	18 (47.4)	20 (52.6)	2.3 (1.1–5.3)	
Stage					
I and II	70 (68.0)	53 (74.6)	18 (25.4)	1.00 (Ref.)	**0.00**
III and IV	33 (32.0)	09 (28.1)	23 (71.9)	7.5 (2.9–19.2)	
Grade					
WD	66 (64.0)	50 (78.1)	14 (21.9)	1.00 (Ref.)	**0.00**
PD	37 (36.0)	12 (30.8)	27 (69.2)	8.1 (3.3–19.8)	

BMI, basal metabolic index (<18.5 = underweight, 18.5–24.99 = Normal, 25–29.99 = Preobese, 30–34.99 = Obese class I, 35–39.99 = Obese class II).

CEA, carcinoembryonic antigen; *H. pylori*, *Helicobacter pylori*; WD, well differentiated; PD, poorly differentiated.

### Association of Relative mRNA Expression With Various Parameters of GC Cases

The stratification of ARID1A mRNA levels with respect to various socio-demographic and clinicopathological parameters of GC patients is given in Table 3. Among GC cases having stage I&II disease only 18.6% were having reduced *ARID1A* mRNA expression compared to 39.4% patients with stage III&IV disease and reduced *ARID1A* mRNA expression (OR = 2.8; *p* = 0.03). A higher percentage (37.9%) of GC patients with poorly differentiated disease were having reduced *ARID1A* mRNA expression compared to GC patients with well differentiated disease (18.2%) (OR = 2.7; *p* = 0.03).

The stratification of TP53 mRNA levels with respect to various socio-demographic and clinicopathological parameters of GC patients is given in Table 4. A significantly higher percentage of Preobese GC patients were having elevated *TP53* mRNA levels compared to GC patients having normal BMI and elevated *TP53* mRNA levels (67.9 vs. 31.5%; OR = 6.3; *p* = 0.0002). Among GC cases without *H. Pylori* infection 35.4% (23/65) were having *TP53* mRNA overexpression compared to GC patients with *H. Pylori* infection among which 68.4% (26/38) were having *TP53* overexpression (OR = 4.0; *p* = 0.002). Among GC cases having stage I & II disease only 38.0% were having reduced *TP53* mRNA expression compared to 68.8% patients with stage III&IV disease and elevated *TP53* mRNA expression (OR = 3.6; *p* = 0.005). A higher percentage of GC patients with poorly differentiated disease were having elevated *TP53* mRNA expression compared to GC patients with well differentiated disease (34.4 vs. 69.2%; OR = 4.3; *p* = 0.001).

The stratification of PDL1 mRNA levels with respect to various socio-demographic and clinicopathological parameters of GC patients is given in Table 5. A significantly higher percentage of GC patients with stage III & IV disease were having elevated PDL1 mRNA levels compared to GC patients having stage I & II disease (71.9 vs. 25.4%; OR = 7.5; *p* = 0.00). Among GC cases with well differentiated disease only 21.9% (14/66) were having *PDL1* mRNA overexpression compared to GC patients with poorly differentiated disease among which 69.2% (27/37) were having *PDL1* overexpression (OR = 8.1; *p* = 0.00).

### Relationship Between ARID1A, TP53 and PDL1 mRNA Expression

**FIGURE 5:**
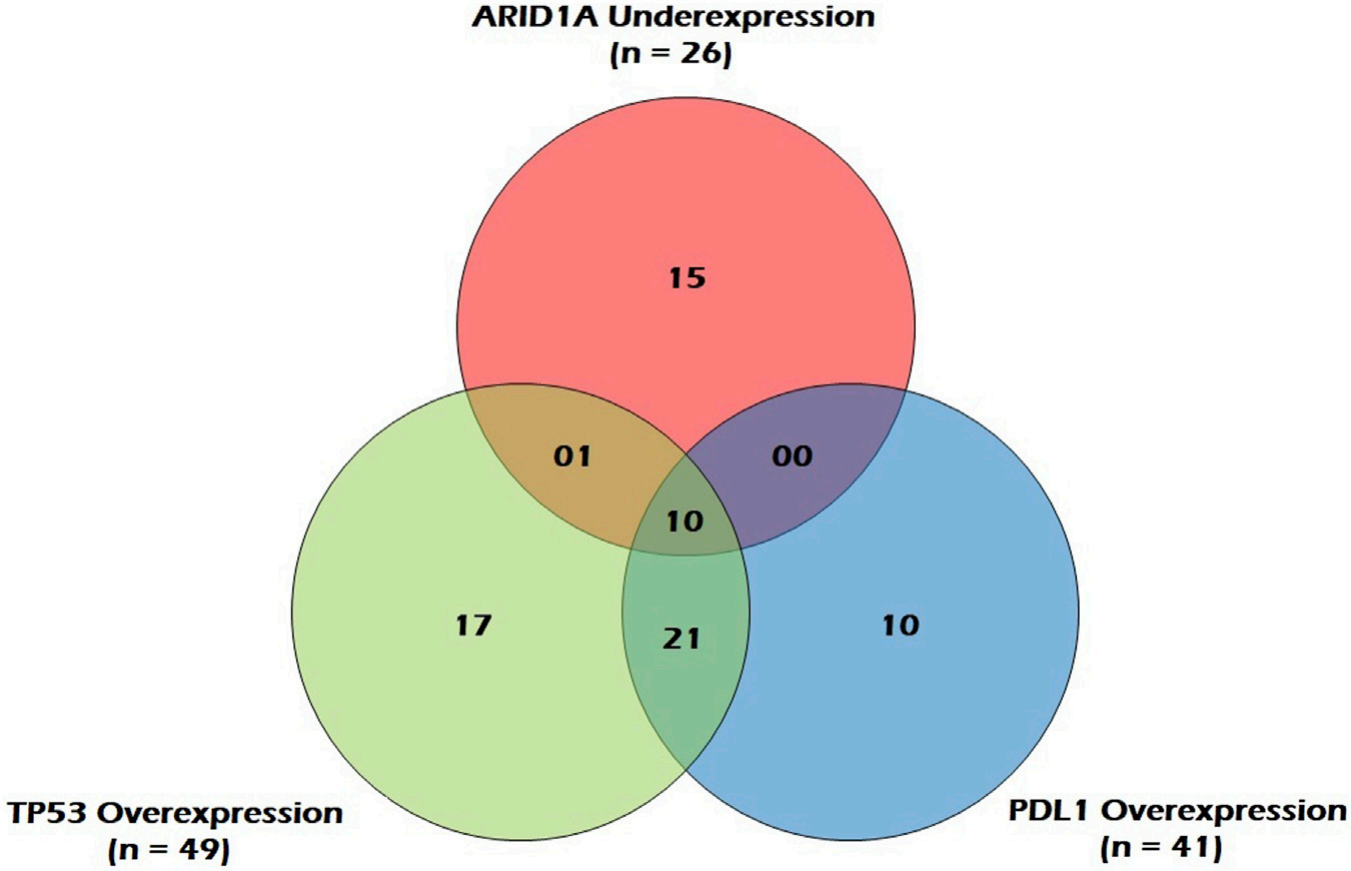
Venn Diagram depicting correlation between ARID1A mRNA underexpression and mRNA overexpression of TP53 and PDL1 in GC patients.

Figure 5 represents a venn diagram depicting the correlation between *ARID1A* mRNA underexpression and mRNA overexpression of *TP53* and *PDL1*. Only *TP53* overexpression and *PDL1* overexpression were significantly associated with each other and the correlation was positive (OR = 7.6; *p* = 0.00; Table 4).

## Discussion

The clinical symptoms of GC are often commenced at an advanced stage, resulting in the limitation of diagnosis and therapeutic approaches to around 50% of cancerous cases [25]. However, over the past decade, there has been a striking improvement in cancer management and treatment by understanding the genetics of disease.

*ARID1A* has recently emerged as a novel tumor suppressor gene, as per the evidence supporting the positive association between reduced *ARID1A* expression and tumorigenicity of several cancers, such as ovarian, endometrial, cervical, breast, gastric, colorectal, and lung cancers [7, 10, 26]. We investigated the status of *ARID1A* mutation in GC wherein we detected a nonsense mutation (c.3219; C > T) among two (∼2.0%) GC patients that introduces a premature stop codon leading to the abortive termination of the ARID1A protein, thereby resulting in its complete or partial inactivation and reduced or loss of protein function. Mamo et al. previously reported nonsense mutation in exon-9, which introduces the premature stop codon into coding sequence at position W1073 [27]. Furthermore, studies have reported that frequency of this nonsense mutation is usually low in tumor cells [27, 28]. However, the general mutation rate of the *ARID1A* gene varies between 8 and 39% in gastrointestinal cancers [29] and between 8 and 29% in GC [26]. Earlier studies have demonstrated that *ARID1A* mutations were more frequent in Gastric tumors, especially with microsatellite instability and Epstein-Barr virus infection [10, 29]. The nonsense mutation of *ARID1A*, resulting in loss of its functional protein, consequently leads to the activation of the PI3K/AKT pathway that promotes several mechanisms responsible for carcinogenesis, including cell proliferation, inhibition of apoptosis, cell adhesion, and transformation [5, 30]. Several studies have reported that the siRNA knockdown of *ARID1A* increases phosphorylations of AKT and promotes cell division and metastasis [5, 30].

Our result revealed that *ARID1A* was under-expressed at mRNA level in 25.6% of GC cases with an average fold change of 0.63. Consistent with our study, Yang *et al.* found that the expressional loss of *ARID1A* was frequent in 30% of GC patients and has a significant correlation with poor survival and prognosis [31]. However, Wang *et al.* revealed that *ARID1A* was significantly lower in 65.15 and 52% of GC patients at mRNA and protein level respectively [10]. Previously it has been reported that about 30% of Caucasians, 25% of Asians and 10% of Pacific Islanders exhibited loss of *ARID1A* expression in clear cell and endometrioid ovarian carcinoma [31]. Additionally, there was no significant difference when comparing individuals of Japanese and non-Japanese origin with respect to their decreased *ARID1A* expression in ovarian cancer (29 vs. 18%) [31]. The expressional loss of *ARID1A* has been shown to trigger the initiation and progression of carcinogenesis in numerous types of cancers, including ovarian, breast, endometrial and cervical, breast, gastric, and colorectal cancers through several mechanisms that have not been fully elucidated [26]. Studies have reported that restoration of normal ARID1A protein levels *in vitro* successfully inhibits the uncontrolled cell division [26]. Chan-on et al. observed that knockdown of ARID1A in three wild-type cell lines promotes the cell division in bile duct cancer, and the effect was reversed when ARID1A was ectopically re-expressed [32]. We further investigated the association of GC with clinicopathological parameters and found lower *ARID1A* expression in patients with the high-grade and advanced stage of the GC. Consistent with our findings, some investigations also reported that the reduced expression of the *ARID1A* has a significant association with higher grading and staging of GC [10]. However, several studies have also reported that loss of ARID1A expression has no significant association with advanced cancer grade and stage [33]. Although not found in our study, Chou et al. revealed that *ARID1A* has a significant association with age, gender, tumor location, and tumor size [34]. Our study confirmed that the mRNA levels of *ARID1A* were low in the two GC patients with *ARID1A* c.3219 C > T mutation. Consistent with our study, Mamo et al. also reported that RNA levels of *ARID1A* were found very low in samples with nonsense mutations [27]. The introduction of stop codon in upstream region results in abortive termination of proteins that could interfere the normal protein functions [35].

The role of *TP53* in suppressing tumour growth is primarily due to its induction of cell cycle arrest and DNA repair or apoptosis, following genotoxic stress [36]. We observed a significantly higher *TP53* relative mRNA expression in 47.6% of GC cases. The mean level of *TP53* mRNA expression was almost 3 fold higher in GC tumours than in adjacent normal mucosa. In our study, some GC tumours showed high *TP53* mRNA levels, while others showed a slight increase. So, *TP53* mRNA may either be weakly expressed in all tumour cells or highly expressed in a few tumour cells owing to tumour heterogeneity. Our results clearly demonstrate that *TP53* regulation may occur at a pretranslational step, involving either an increase in *TP53* gene expression and/or stabilization of its mRNA which might lead to elevated content of TP53 protein in the cell. Although there is a particular paucity of studies that have analysed the relative expression of *TP53* mRNA in cancer, *TP53* has been shown to express at a high frequency in gastric adenocarcinomas [37]. In line with our observation *TP53* mRNA expression was significantly higher in triple-negative breast cancer (TNBC) [38]. Our findings confirm earlier reports, which showed an elevated level of *TP53* transcripts in 70% [39] and 66% [40] of tested CRC tumours respectively. Overexpression of *TP53* mRNA has recently been shown to increase the amount of endogenous TP53 and to increase apoptosis in human melanoma cells, in part, by modulating the transcription of downstream target genes including downregulation of p21 and upregulation of *TP53*-induced death domain protein, to favour apoptosis rather than cell cycle arrest [41]. Reports suggest that overexpressed mRNA can enhance or inhibit the ability of TP53 to trans activate certain target promoters and to induce apoptosis [42]. Thus, regulated expression of TP53 isoforms is critical for the biological outcome of TP53.

On stratification, we observed a significant association of elevated *TP53* mRNA levels with higher stage and higher grade of GC. In tune with our observations, Fenoglio-Preiser *et al.* has reported *TP53* overexpression in almost 90% of invasive Gastric tumors [43]. Increased expression of *TP53* mRNA in ovarian and renal cell carcinoma has been associated with worse prognosis and higher tumour grade [44]. As per previous studies, the degree of TP53 expression correlates positively with the proliferative rate of the tumors [45] and there is a tendency for *TP53* expression to be more common in poorly differentiated tumors than in well differentiated lesions [46]. Kakeji et al. [47] showed that tumors with TP53-positive staining had a higher proliferative activity than did those that stained negative. Previously, breast cancer tumors of highly malignant potential and poor prognosis showed higher expression of TP53 protein [21]. In contradiction with our study, *TP53* mRNA overexpression was associated with lower recurrence rates and higher overall survival rates in breast cancer and gastric cancer [37, 48]. In addition, no correlation was found between *TP53* mRNA, tumour stage and disease prognosis in CRC and GC [49, 50]. Similarly, as per few previous studies, no correlation was found between positive TP53 tissue status and histological grade of tumor differentiation [51]. It has been suggested that *TP53* mRNA also harbours information that helps control TP53 protein turnover rate [52].

In our study, most of the GC patients with *H. Pylori* infection had significantly increased *TP53* mRNA levels. Shiao et al. observed overexpression of *TP53* in 15% of *H. Pylori*–positive chronic gastritis patients but 38% of *H. Pylori*–positive metaplastic gastritis patients [53]. It has been confirmed that infection from *H. Pylori* is a major cause of chronic inflammation of the human gastric antral mucosa leading to development of atrophic chronic gastritis and Gastric carcinoma (CG) [54]. In consonance with our study, it has been shown that *H. Pylori* infection increased *TP53* expression and the apoptosis rate in GC [55]. Wei et al. found that *TP53* levels before *H. Pylori* infection were low or undetectable and were elevated on exposure to *H. Pylori* infection accompanied with intense inflammation [56]. According to a study by Ahmed et al. cells cultivated with *H. Pylori* were found to be in phase G1 of the cell cycle with *TP53* overexpression suggesting that cell cycle arrest in G1 is associated with a reduction in cyclin E levels and an increase in TP53 and p21 expression showing that *H. Pylori* can induce cell stress, reduce the ability to repair damaged cells, and can increase the number of changes in the genome, leading to genetic instability and finally to GC [57].

PD1 acts as a T-cells inhibitor mainly by limiting T-cells activity within neoplastic tissues and its ligand, PDL1, is often overexpressed on tumor cells [18]. In our study, *PDL1* relative mRNA expression was significantly higher in 39.8% of GC cases with an average fold change of 2.43 in tumor tissues compared to adjacent normal tissues. Wu et al. showed that immunohistochemical PDL1 expression was strongly positive in 42.2% of 102 human gastric carcinomas, weakly positive in adenoma samples and totally negative in normal gastric tissue [58]. PDL1 expression has been reported in a wide variety of solid tumors, including lung cancer, hepatocellular carcinoma and intra-hepatic cholangiocarcinoma, gastric, colorectal, pancreatic, ovarian, breast, cervical and oral cancer, head and neck squamous cell carcinomas, nasopharyngeal, esophageal, urothelial and renal cell cancer, nephroblastoma, melanoma and gliomas [58, 59]. It has been suggested that CD8^+^ T cells upregulate PD1 expression and secrete IFN-γ when they encounter tumor antigens, resulting in the upregulation of *PDL1* expression on tumor cells and immune cells and the ligation of PDL1 with PD1 will decrease T cell function and create a negative feedback mechanism that decreases antitumor immunity leading to tumorigenesis [22]. In contradiction with our observation, no statistically significant differences were found with regard to PDL1 mRNA levels within normal and GC specimens as previously verified by Chen et al. [60].

On stratification, we observed a significant association of elevated *PDL1* mRNA levels with higher stage and higher grade of GC. *PDL1* overexpression has been associated to higher number of lymph node metastasis, larger tumor size, increased depth of invasion and poorer overall survival in various cancers [58, 61]. According to a previous study, *PD1, PDL1* and *CD8* mRNA levels were significantly higher in undifferentiated GC [22]. More recently, it was demonstrated that PDL1 overexpression was a worse prognostic factor in GC [19].

In our study, we observed a positive correlation between *TP53* and *PDL1* mRNA expression, suggesting that there is a synergistic effect between *PDL1* and *TP53* in the occurrence and development of tumors, which has also been demonstrated in NSCLC wherein *TP53* has been shown to regulate *PDL1* expression *via* miR-34 that binds PDL1 3′-untranslated region in NSCLC models [62, 63]. Moreover, the expression of *PDL1* and *TP53* has previously been positively correlated [64]. All these studies including ours’ linked tumor immune evasion to other tumor suppressor pathways previously described for TP53 [65].

## Conclusion

In summary, we observed down regulation of *ARID1A* mRNA expression and upregulation of *TP53* and *PDL1* mRNA expression in GC which was in turn significantly associated high-grade and advanced stage of tumor suggesting that lower *ARID1A* expression and higher expression of *TP53* and *PDL1* might play a definite role in the initiation and progression of GC. Furthermore, a positive correlation was found between *TP53* and *PDL1* mRNA expression. This should be useful for future anti-tumour research and for the design of therapeutic agents specific to the inactivation process. However, further largescale and comprehensive researches are needed to support our results and conclusion.

## Data Availability

The data will be made available upon reasonable request.

## References

[1] Sung H Ferlay J Siegel RL Laversanne M Soerjomataram I Jemal A Global Cancer Statistics 2020: GLOBOCAN Estimates of Incidence and Mortality Worldwide for 36 Cancers in 185 Countries. CA A Cancer J Clin (2021) 71:209–49. 10.3322/caac.21660 33538338

[2] Qurieshi MA Khan SMS Masoodi MA Qurieshi U Ain Q Jan Y Epidemiology of Cancers in Kashmir, India: an Analysis of Hospital Data. Adv Prev Med (2016) 2016:1–6. 10.1155/2016/1896761 PMC4949346 27478644

[3] Oki E Kakeji Y Zhao Y Yoshida R Ando K Masuda T Chemosensitivity and Survival in Gastric Cancer Patients with Microsatellite Instability. Ann Surg Oncol (2009) 16:2510–5. 10.1245/s10434-009-0580-8 19565284

[4] Wilson BG Roberts CWM. SWI/SNF Nucleosome Remodellers and Cancer. Nat Rev Cancer (2011) 11:481–92. 10.1038/nrc3068 21654818

[5] Samartzis E Noske A Dedes K Fink D Imesch P. ARID1A Mutations and PI3K/AKT Pathway Alterations in Endometriosis and Endometriosis-Associated Ovarian Carcinomas. Int J Mol Sci (2013) 14(9):18824–49. 10.3390/ijms140918824 24036443 PMC3794809

[6] Qadir J Majid S Khan MS Rashid F Wani MD Din I AT-rich Interaction Domain 1A Gene Variations: Genetic Associations and Susceptibility to Gastric Cancer Risk. Pathol Oncol Res (2020) 26(4):2237–46. 10.1007/s12253-020-00815-1 32377988

[7] Mathur R Roberts CWM. SWI/SNF (BAF) Complexes: Guardians of the Epigenome. Annu Rev Cancer Biol (2018) 2:413–27. 10.1146/annurev-cancerbio-030617-050151

[8] Jones S Li M Parsons DW Zhang X Wesseling J Kristel P Somatic Mutations in the Chromatin Remodeling Gene ARID1A Occur in Several Tumor Types. Hum Mutat (2012) 33(1):100–3. 10.1002/humu.21633 22009941 PMC3240719

[9] Abe H Maeda D Hino R Otake Y Isogai M Ushiku AS ARID1A Expression Loss in Gastric Cancer: Pathway-dependent Roles with and without Epstein-Barr Virus Infection and Microsatellite Instability. Virchows Arch (2012) 461:367–77. 10.1007/s00428-012-1303-2 22915242

[10] Wang D-d. Chen Y-b. Pan K Wang W Chen S-p. Chen J-g. Decreased Expression of the ARID1A Gene Is Associated with Poor Prognosis in Primary Gastric Cancer. PLoS One (2012) 7:e40364. 10.1371/journal.pone.0040364 22808142 PMC3396657

[11] Levine AJ. p53, the Cellular Gatekeeper for Growth and Division. Cell (1997) 88:323–31. 10.1016/s0092-8674(00)81871-1 9039259

[12] Ho L Crabtree GR. Chromatin Remodelling during Development. Nature (2010) 463:474–84. 10.1038/nature08911 20110991 PMC3060774

[13] Bosse T ter Haar NT Seeber LM Diest PJv. Hes FJ Vasen HF Loss of ARID1A Expression and its Relationship with PI3K-Akt Pathway Alterations, TP53 and Microsatellite Instability in Endometrial Cancer. Mod Pathol (2013) 26:1525–35. 10.1038/modpathol.2013.96 23702729

[14] Hanahan D Weinberg RA. Hallmarks of Cancer: the Next Generation. Cell (2011) 144:646–74. 10.1016/j.cell.2011.02.013 21376230

[15] Hou J Yu Z Xiang R Li C Wang L Chen S Correlation between Infiltration of FOXP3+ Regulatory T Cells and Expression of B7-H1 in the Tumor Tissues of Gastric Cancer. Exp Mol Pathol (2014) 96:284–91. 10.1016/j.yexmp.2014.03.005 24657498

[16] Naito T Udagawa H Umemura S Sakai T Zenke Y Kirita K Non-small Cell Lung Cancer with Loss of Expression of the SWI/SNF Complex Is Associated with Aggressive Clinicopathological Features, PD-L1-Positive Status, and High Tumor Mutation burden. Lung Cancer (2019) 138:35–42. 10.1016/j.lungcan.2019.10.009 31630044

[17] Kim YB Ahn JM Bae WJ Sung CO Lee D. Functional Loss of ARID1A Is Tightly Associated with High PD‐L1 Expression in Gastric Cancer. Int J Cancer (2019) 145:916–26. 10.1002/ijc.32140 30664822

[18] Silva R Gullo I Carneiro F. The PD-1:PD-L1 Immune Inhibitory Checkpoint in *Helicobacter pylori* Infection and Gastric Cancer: a Comprehensive Review and Future Perspectives. Porto Biomed J (2016) 1(1):4–11. 10.1016/j.pbj.2016.03.004 32258540 PMC6806944

[19] Gu L Chen M Guo D Zhu H Zhang W Pan J PD-L1 and Gastric Cancer Prognosis: A Systematic Review and Meta-Analysis. PLoS One (2017) 12(8):e0182692. 10.1371/journal.pone.0182692 28796808 PMC5552131

[20] Waddell T Chau I Cunningham D Gonzalez D Okines AFC Wotherspoon A Epirubicin, Oxaliplatin, and Capecitabine with or without Panitumumab for Patients with Previously Untreated Advanced Oesophagogastric Cancer (REAL3): a Randomised, Open-Label Phase 3 Trial. Lancet Oncol (2013) 14:481–9. 10.1016/s1470-2045(13)70096-2 23594787 PMC3669518

[21] Liu H Zhou M. Evaluation of P53 Gene Expression and Prognosis Characteristics in Uveal Melanoma Cases. Onco Targets Ther (2017) 10:3429–34. 10.2147/ott.s136785 28744147 PMC5513883

[22] Ito S Masuda T Noda M Hu Q Shimizu D Kuroda Y Prognostic Significance of PD-1, PD-L1 and CD8 Gene Expression Levels in Gastric Cancer. Oncology (2020) 98(7):501–11. 10.1159/000506075 32380498

[23] Livak KJ Schmittgen TD. Analysis of Relative Gene Expression Data Using Real-Time Quantitative PCR and the 2−ΔΔCT Method. Methods (2001) 25(4):402–8. 10.1006/meth.2001.1262 11846609

[24] Hu N Qian L Hu Y Shou J-Z Wang C Giffen C Quantitative Real-Time RT-PCR Validation of Differential mRNA Expression of SPARC, FADD, Fascin, COL7A1, CK4, TGM3, ECM1, PPL and EVPLin Esophageal Squamous Cell Carcinoma. BMC Cancer (2006) 6:33. 10.1186/1471-2407-6-33

[25] Nobili S Bruno L Landini I Napoli C Bechi P Tonelli F Genomic and Genetic Alterations Influence the Progression of Gastric Cancer. World J Gastroenterol (2011) 17(3):290–9. 10.3748/wjg.v17.i3.290 21253387 PMC3022288

[26] Pavlidou EN Balis V. Diagnostic Significance and Prognostic Role of the ARID1A Gene in Cancer Outcomes (Review). World Acad Sci J (2020) 2:49–64. 10.3892/wasj.2020.37

[27] Mamo A Cavallone L Tuzmen S Chabot C Ferrario C Hassan S An Integrated Genomic Approach Identifies ARID1A as a Candidate Tumor-Suppressor Gene in Breast Cancer. Oncogene (2012) 31(16):2090–100. 10.1038/onc.2011.386 21892209

[28] Sjöblom T Jones S Wood LD Parsons DW Lin J Barber TD The Consensus Coding Sequences of Human Breast and Colorectal Cancers. Science (2006) 314(5797):268–74. 10.1126/science.1133427 16959974

[29] Erfani M Hosseini SV Mokhtari M Zamani M Tahmasebi K Alizadeh Naini M Altered ARID1A Expression in Colorectal Cancer. BMC Cancer (2020) 20(1):350. 10.1186/s12885-020-6706-x 32334542 PMC7183124

[30] Yang Y Wang X Yang J Duan J Wu Z Yang F Loss of ARID1A Promotes Proliferation, Migration and Invasion via the Akt Signaling Pathway in NPC. Cancer Manag Res (2019) 11:4931–46. 10.2147/cmar.s207329 31213911 PMC6549766

[31] Lai T Vierkoetter K Ayabe A Jun Ahn H Shimizu D Keith YT. Ethnic Variations in ARID1a Expression in clear Cell and Endometrioid Ovarian Carcinoma. J Clin Oncol (2016) 34(15Suppl. l):e17073. 10.1200/jco.2016.34.15_suppl.e17073

[32] Chan-On W Nairismägi M-L Ong CK Lim WK Dima S Pairojkul C Exome Sequencing Identifies Distinct Mutational Patterns in Liver Fluke-Related and Non-infection-related Bile Duct Cancers. Nat Genet (2013) 45(12):1474–8. 10.1038/ng.2806 24185513

[33] Lee SY Kim D-W Lee HS Ihn MH Oh H-K Park DJ Loss of AT-Rich Interactive Domain 1A Expression in Gastrointestinal Malignancies. Oncology (2015) 88(4):234–40. 10.1159/000369140 25503393

[34] Chou A Toon CW Clarkson A Sioson L Houang M Watson N Loss of ARID1A Expression in Colorectal Carcinoma Is Strongly Associated with Mismatch Repair Deficiency. Hum Pathol (2014) 45(8):1697–703. 10.1016/j.humpath.2014.04.009 24925223

[35] Jopling CL. Stop that Nonsense!!. eLife (2014) 3:e04300. 10.7554/eLife.04300 25205670 PMC4155323

[36] Olivier M Hollstein M Hainaut P. TP53 Mutations in Human Cancers: Origins, Consequences, and Clinical Use. Cold Spring Harbor Perspect Biol (2010) 2:a001008. 10.1101/cshperspect.a001008 PMC2827900 20182602

[37] Ogawa M Onoda N Maeda K Kato Y Nakata B Kang S-M A Combination Analysis of P53 and P21 in Gastric Carcinoma as a strong Indicator for Prognosis. Int J Mol Med (2001) 7:479–83. 10.3892/ijmm.7.5.479 11295107

[38] Avery-Kiejda KA Morten B Wong-Brown MW Mathe A Scott RJ. The Relative mRNA Expression of P53 Isoforms in Breast Cancer Is Associated with Clinical Features and Outcome. Carcinogenesis (2014) 35(3):586–96. 10.1093/carcin/bgt411 24336193

[39] Gope ML Chun M Gope R. Comparative Study of the Expression of Rb and P53 Genes in Human Colorectal Cancers, colon Carcinoma Cell Lines and Synchronized Human Fibroblasts. Mol Cel Biochem (1991) 107:55–63. 10.1007/bf02424576 1784274

[40] Lothe RA Fossli T Danielsen HE Stenwig AE Nesland JM Gallie B Molecular Genetic Studies of Tumor Suppressor Gene Regions on Chromosomes 13 and 17 in Colorectal Tumors. JNCI J Natl Cancer Inst (1992) 84:1100–8. 10.1093/jnci/84.14.1100 1619684

[41] Takahashi R Markovic SN Scrable HJ. Dominant Effects of Δ40p53 on P53 Function and Melanoma Cell Fate. J Invest Dermatol (2014) 134(3):791–800. 10.1038/jid.2013.391 24037342 PMC3945389

[42] Bourdon J-C Fernandes K Murray-Zmijewski F Liu G Diot A Xirodimas DP p53 Isoforms Can Regulate P53 Transcriptional Activity. Genes Dev (2005) 19:2122–37. 10.1101/gad.1339905 16131611 PMC1221884

[43] Fenoglio-Preiser CM Wang J Stemmermann GN Noffsinger A. TP53 and Gastric Carcinoma: a Review. Hum Mutat (2003) 21(3):258–70. 10.1002/humu.10180 12619111

[44] Hofstetter G Berger A Fiegl H Slade N Zorić A Holzer B Alternative Splicing of P53 and P73: the Novel P53 Splice Variant P53δ Is an Independent Prognostic Marker in Ovarian Cancer. Oncogene (2010) 29:1997–2004. 10.1038/onc.2009.482 20101229

[45] Ioachim E Goussia A Stefanou D Agnantis NJ. Expression of P53 Protein in Gastric Cancer: an Immunohistochemical Study with Correlation to Proliferative Activity. Anticancer Res (1997) 17:513–7. 9066704

[46] Sasaki I Yao T Nawata H Tsuneyoshi M. Minute Gastric Carcinoma of Differentiated Type with Special Reference to the Significance of Intestinal Metaplasia, Proliferative Zone, and P53 Protein during Tumor Development. Cancer (1999) 85:1719–29. 10.1002/(sici)1097-0142(19990415)85:8<1719:aid-cncr11>3.0.co;2-v 10223565

[47] Kakeji Y Korenaga D Tsujitani S Baba H Anai H Maehara Y Gastric Cancer with P53 Overexpression Has High Potential for Metastasising to Lymph Nodes. Br J Cancer (1993) 67:589–93. 10.1038/bjc.1993.108 8439509 PMC1968266

[48] Mönig SP Eidt S Zirbes TK Stippel D Baldus SE Pichlmaier H. p53 Expression in Gastric Cancer: Clinicopathological Correlation and Prognostic Significance. Dig Dis Sci (1997) 42:2463–7. 10.1023/a:1018844008068 9440620

[49] El-Mahdani N Vaillant J-C Guiguet M Prévot S Bertrand V Bernard C Overexpression of P53 mRNA in Colorectal Cancer and its Relationship to P53 Gene Mutation. Br J Cancer (1997) 75(4):528–36. 10.1038/bjc.1997.92 9052405 PMC2063311

[50] Mühlmann G Öfner D Zitt M Müller HM Maier H Moser P 14-3-3 Sigma and P53 Expression in Gastric Cancer and its Clinical Applications. Dis Markers (2010) 29(1):21–9. 10.1155/2010/470314 20826914 PMC3835372

[51] Carneiro F David L Sobrinho-Simoes M Seruca R Nesland JM. Oncogene S and Onco-Suppressor Genes in Gastric Cancer Carcinoma. Surg Pathol (1994) 5(3):225–38.

[52] Haronikova L Olivares-Illana V Wang L Karakostis K Chen S Fahraeus R. The P53 mRNA: an Integral Part of the Cellular Stress Response. Nucleic Acids Res (2019) 47(7):3257–71. 10.1093/nar/gkz124 30828720 PMC6468297

[53] Shiao YH Rugge M Correa P Lehmann HP Scheer WD. p53 Alteration in Gastric Precancerous Lesions. Am J Pathol (1994) 144:511–7. 8129036 PMC1887105

[54] Misiewicz JJ. The Sydney System: A New Classification of Gastritis. Introduction. J Gastroenterol Hepatol (1991) 6(3):207–8. 10.1111/j.1440-1746.1991.tb01467.x 1912430

[55] Morales-Fuentes GA Zarate-Osorno A Quiñónez-Urrego EE Antonio-Manrique M Martínez-García CL Figueroa-Barojas P p53 expresado en la mucosa gástrica de pacientes infectados por *Helicobacter pylori* . Rev Gastroenterol Méx (2013) 78(1):12–20. 10.1016/j.rgmx.2012.11.001 23374541

[56] Wei J Nagy TA Vilgelm A Zaika E Ogden SR Romero–Gallo J Regulation of P53 Tumor Suppressor by *Helicobacter pylori* in Gastric Epithelial Cells. Gastroenterology (2010) 139:1333–43. 10.1053/j.gastro.2010.06.018 20547161 PMC2949494

[57] Ahmed A Smoot D Littleton G Tackey R Walters CS Kashanchi F *Helicobacter pylori* Inhibits Gastric Cell Cycle Progression. Microbes Infect (2000) 2:1159–69. 10.1016/s1286-4579(00)01270-3 11008106

[58] Wu C Zhu Y Jiang J Zhao J Zhang X-G Xu N. Immunohistochemical Localization of Programmed Death-1 Ligand-1 (PD-L1) in Gastric Carcinoma and its Clinical Significance. Acta Histochem (2006) 108:19–24. 10.1016/j.acthis.2006.01.003 16530813

[59] Sun J Xu K Wu C Wang Y Hu Y Zhu Y PD-L1 Expression Analysis in Gastric Carcinoma Tissue and Blocking of Tumor-Associated PD-L1 Signaling by Two Functional Monoclonal Antibodies. Tissue Antigens (2007) 69:19–27. 10.1111/j.1399-0039.2006.00701.x 17212704

[60] Chen XL Cao XD Kang AJ Wang KM Su BS Wang YL. *In Situ* expression and Significance of B7 Costimulatory Molecules within Tissues of Human Gastric Carcinoma. World J Gastroenterol (2003) 9:1370–3. 10.3748/wjg.v9.i6.1370 12800259 PMC4611819

[61] Afreen S Dermime S. The Immunoinhibitory B7-H1 Molecule as a Potential Target in Cancer: Killing many Birds with One Stone. Hematol Oncol Stem Cel Ther (2014) 7:1–17. 10.1016/j.hemonc.2013.09.005 24398144

[62] Cortez MA Ivan C Valdecanas D Wang X Peltier HJ Ye Y PDL1 Regulation by P53 via miR-34. J Natl Cancer Inst (2016) 108(1):djv303. 10.1093/jnci/djv303 26577528 PMC4862407

[63] Cha YJ Kim HR Lee CY Cho BC Shim HS. Clinicopathological and Prognostic Significance of Programmed Cell Death Ligand-1 Expression in Lung Adenocarcinoma and its Relationship with P53 Status. Lung Cancer (2016) 97:73–80. 10.1016/j.lungcan.2016.05.001 27237031

[64] Tojyo I Shintani Y Nakanishi T Okamoto K Hiraishi Y Fujita S PD-L1 Expression Correlated with P53 Expression in Oral Squamous Cell Carcinoma. Maxillofac Plast Reconstr Surg (2019) 41(1):56. 10.1186/s40902-019-0239-8 31857991 PMC6892985

[65] Chang T-C Wentzel EA Kent OA Ramachandran K Mullendore M Lee KH Transactivation of miR-34a by P53 Broadly Influences Gene Expression and Promotes Apoptosis. Mol Cel (2007) 26:745–52. 10.1016/j.molcel.2007.05.010 PMC1939978 17540599

